# PEACE: Development and Validation of a Brief Five-Item Sleep Quality Scale for Community and Primary Care

**DOI:** 10.3390/medicina62040757

**Published:** 2026-04-15

**Authors:** Giuseppe Di Lorenzo, Luca Scafuri, Francesco Passaro, Raffaele Baio, Eleonora Monteleone, Vittorio Riccio, Luigia Maglione, Andrea Torcia, Paola Tarantino, Armando Calogero, Antonio Ruffo, Filippo Varlese, Michele Musone, Ciro Imbimbo, Luigi De Luca, Giuseppe Romeo, Francesco Stanzione, Rossella Di Trolio, Oriana Strianese, Raffaele Balsamo, Lorenzo Spirito, Antonio Reia, Gabriele Barbato, Sisto Perdonà, Francesca Cappuccio, Carlo Buonerba, Felice Crocetto

**Affiliations:** 1 Oncology Unit, “Andrea Tortora” Hospital, ASL Salerno, 84016 Pagani, Italy; 2 Associazione O.R.A. ETS—Oncology Research Assistance, 84134 Salerno, Italy; 3 Department of Medicine, Saint Camillus International University of Health and Medical Sciences (UniCamillus), 00131 Rome, Italy; 4 Department of Urology, Istituto Nazionale Tumori IRCCS “Fondazione G. Pascale”, 80131 Naples, Italy; 5 Department of Urology, Presidio Ospedaliero Umberto I, 84014 Nocera Inferiore, Italy; 6 Ospedale S. Maria della Pietà (Camilliani), 80026 Casoria, Italy; 7 U.O.C. Lungodegenza (Long-Term Care Unit), P.O. “Santissima Maria della Pietà” Hospital, 80026 Casoria, Italy; 8 Department of Anesthesia, Critical Care and Pain Medicine, Hospital General Universitario de Castellón, 12004 Castelló de la Plana, Spain; 9 Department of Medicine and Health Sciences “Vincenzo Tiberio”, University of Molise, 86039 Termoli, Italy; 10 Department of Advanced Biomedical Sciences, University of Naples “Federico II”, 80131 Naples, Italy; 11 Urology Unit, Department of Neurosciences, Reproductive and Odontostomatological Sciences, University of Naples “Federico II”, 80131 Naples, Italy; 12 Department of Urology, A.O.R.N. Antonio Cardarelli Hospital, Via A. Cardarelli, 9, 80131 Naples, Italy; 13 UOC of General, Emergency, Oncologic and Endocrine Surgery, Pineta Grande Hospital, 81030 Castel Volturno, Italy; 14 Unit of Melanoma, Cancer Immunotherapy and Development Therapeutics, Istituto Nazionale Tumori IRCCS “Fondazione G. Pascale”, 80131 Naples, Italy; 15 UOC Urology, Department of General and Specialized Surgery, Monaldi Hospital—A.O.R.N. Ospedali dei Colli, 80131 Naples, Italy; 16 Urology Unit, Department of Woman, Child and General and Specialized Surgery, University of Campania “Luigi Vanvitelli”, 80131 Naples, Italy; 17 UOC Neurologia—Stroke Unit, Presidio Ospedaliero “San Giuseppe Moscati”, ASL Caserta, Via Antonio Gramsci, 1, 81031 Aversa, Italy; 18 Department of Anesthesia and Intensive Care, Presidio Ospedaliero “Anastasia Guerriero”, ASL Caserta, Viale Sossietta Scialla, 81025 Marcianise, Italy

**Keywords:** sleep quality, patient-reported outcome, primary care, community-dwelling adults, fatigue, well-being

## Abstract

*Background and Objectives*: Poor sleep is common in community and primary-care settings, yet very brief sleep measures suitable for routine use remain limited. We developed and evaluated the five-item Promoting Evaluation and Awareness of Comfort in Sleep (PEACE) scale and examined its associations with well-being and fatigue. *Materials and Methods*: In a cross-sectional, clinician-mediated online survey, 312 community-dwelling adults in Italy who were not receiving active treatment for major diseases completed PEACE, the World Health Organization-Five Well-Being Index (WHO-5), and a short fatigue questionnaire. The sample was stratified and split into exploratory and confirmatory subsamples for factor analyses. *Results*: Factor analyses supported the use of a single total score and showed acceptable reliability. Results were broadly similar in women and men, with no evidence of item-level bias, although some model-comparison indices were mixed. Higher PEACE scores were associated with better well-being and lower fatigue. Adding PEACE to a model predicting well-being from body mass index and sex increased explained variance from 4.0% to 11.5%. *Conclusions*: PEACE is a brief sleep-quality measure with promising initial psychometric properties. In this sample, it was associated with well-being and fatigue and may add information beyond body mass index and sex in community and primary-care settings.

## 1. Introduction

Sleep quality is increasingly recognized as a core domain of health, with clear implications for cardiometabolic risk, mental health, daytime functioning and quality of life [1,2,3,4]. Poor or fragmented sleep is associated with adverse metabolic and cardiovascular profiles, mood disturbance, impaired cognition and reduced functional capacity, whereas good sleep supports emotional regulation, functioning and safety [1,2,3,4]. Recent public-health statements therefore argue that sleep health should be promoted alongside diet, physical activity and healthy weight as a modifiable determinant of population health [2,4].

In everyday clinical practice, complaints related to poor sleep are common in both general-medicine and specialist settings and often coexist with low mood, anxiety and fatigue. Generic well-being indices such as the World Health Organization–Five Well-Being Index (WHO-5) capture cheerfulness, vitality, interest and feeling rested, all of which may be influenced by sleep [5]. Capturing perceived sleep quality alongside well-being and fatigue is therefore informative in community and primary-care samples.

Several patient-reported outcome measures (PROMs) are available to assess sleep. The Pittsburgh Sleep Quality Index (PSQI) remains the most widely used global sleep-quality instrument, but its length and component scoring may be impractical for routine primary care [6]. Other options include PROMIS sleep-disturbance instruments [7,8], the Sleep Condition Indicator [9], the Jenkins Sleep Scale [10] and the Insomnia Severity Index [11]. However, there remains a need for a very brief stand-alone sleep-quality score that is easy to administer and interpret alongside generic outcomes such as well-being and fatigue.

Within this context, there is a clear need for a concise sleep-quality PROM that preserves validity while remaining feasible for community, primary-care and lifestyle-medicine applications [2,4].

Any new instrument intended for clinical and epidemiological research should meet contemporary standards for PROM development and evaluation, including clear content definition and evidence of content validity, structural validity, reliability and invariance [12,13].

To address these gaps, two co-authors (CB and GDL) developed the Promoting Evaluation and Awareness of Comfort in Sleep (PEACE) questionnaire, a five-item patient-reported scale created through multidisciplinary consensus to prioritize essential content and feasibility. PEACE assesses core facets of sleep health—sleep duration, subjective sleep quality and sleep continuity (difficulty initiating sleep, nocturnal awakenings and early-morning awakening)—over a 30-day recall period using ordered 0–4 response options, yielding a total score from 0 to 20 (higher scores indicating better sleep).

A prior report from the PREVES-STOP study provided an initial psychometric evaluation of three tools, including PEACE, among 88 participants [14]. The present manuscript is a distinct and substantially expanded validation focused specifically on PEACE in 312 community-dwelling adults and adds formal content validity assessment, split-sample exploratory and confirmatory factor analyses, measurement invariance testing, differential item functioning analyses and incremental validity analyses.

In this prospectively planned cross-sectional study of community-dwelling Italian adults not undergoing treatment for major diseases, we sought to evaluate PEACE as a brief sleep-quality PROM suitable for general-medicine and primary-care contexts. Specifically, our objectives were to (i) examine the scale’s content and construct validity, internal consistency and factorial structure, including measurement invariance by sex; (ii) assess convergent validity with psychological well-being using WHO-5 [5] and related-but-distinct associations with fatigue; and (iii) test whether PEACE contributes incremental information about well-being beyond basic covariates such as body-mass index (BMI) and sex.

## 2. Materials and Methods

### 2.1. Study Design and Participants

We conducted a cross-sectional, anonymous online survey targeting community-dwelling adults (≥18 years) residing in Italy. The study was promoted by Associazione ORA ETS (www.oncologiaora.it, accessed on 10 October 2024) as a not-for-profit initiative. The protocol was approved by the institutional ethics committee (CE/2024/002, 10 October 2024), and the study was conducted in accordance with the Declaration of Helsinki.

Before any study activity, prospective participants reviewed an electronic information sheet and provided electronic informed consent. The information sheet stated that participation was voluntary and anonymous, explained the study purpose and the research use of de-identified questionnaire responses, and indicated that the study involved questionnaire completion only.

Recruitment was clinician-mediated and embedded within routine care pathways. Collaborating healthcare professionals, including general practitioners and specialists from various disciplines, identified potentially eligible adults during standard consultations (including caregivers and accompanying persons) and invited them to participate by scanning a QR code with their smartphone, which directed them to a Google Forms questionnaire. Eligibility, adjudicated by the attending physician, required the capacity to complete the online survey independently and the absence of major diseases under active treatment at the time of participation. Because invitations were delivered opportunistically during routine consultations and access was QR-based, the number of adults approached and the number who declined were not recorded; accordingly, a formal response rate could not be estimated. This clinician-gateway, QR-enabled recruitment and physician-adjudicated eligibility process defined the final analytic sample.

### 2.2. Measures

The online questionnaire included the five PEACE sleep items as the primary sleep-specific measure, together with two brief generic PROMs: the WHO-5 Well-Being Index [5] and a short fatigue scale previously applied in the PREVES-STOP study [14].

PEACE comprises five items scored 0–4 over a 30-day recall:

PEACE-1: Sleep duration

PEACE-2: Subjective sleep quality

PEACE-3: Sleep-onset difficulty

PEACE-4: Night-time awakenings

PEACE-5: Early-morning awakening

Higher scores indicate better sleep (total 0–20). Table 1 reports literal English translations of the administered Italian items.

WHO-5 is a five-item index of positive mental well-being, covering cheerfulness, relaxation, vitality, waking feeling fresh and rested, and interest in daily activities over the preceding two weeks [5]. Each item is rated from 0 (“at no time”) to 5 (“all of the time”), yielding a total score from 0 (worst) to 25 (best possible well-being), which can be rescaled to 0–100 by multiplying by 4. Lower scores reflect poorer mental well-being; in this study, we analyzed the 0–25 raw score (higher values = better well-being) and treated WHO-5 as our main indicator of psychological well-being.

REST is a short fatigue scale operationalised in previous work [14]. In this study, it served as an external anchor to examine the relationship between sleep quality and perceived tiredness.

Sociodemographic and clinical variables included age, sex, educational attainment, BMI and self-reported comorbidities.

### 2.3. Content Validity

Following COSMIN guidance, we conducted a structured content validity study to evaluate the PEACE items for comprehensibility, comprehensiveness and relevance [12,13]. Adults were recruited from the same community and primary-care settings as the main survey via clinician-mediated invitations with QR-code access under protocol CE/2024/002; electronic informed consent was obtained.

Cognitive interviewing used a think-aloud approach with neutral probes targeting comprehension, retrieval, judgement and response mapping. Interviews were conducted in two planned waves (n = 10 per wave). Wave 1 focused on open think-aloud responses, with a pre-specified stopping rule (practical saturation defined as ≥3 consecutive interviews without new meaning-level problems for any item). Wave 2 used structured debriefing to confirm understanding across underrepresented subgroups. Interviewers summarized notes into an item-by-interview problem matrix with severity tags (none/low/moderate/high).

In parallel, a six-member expert panel (oncology, general practice, neuropsychiatry, psychology and pulmonology) independently rated each item on 4-point scales for relevance (primary), clarity and simplicity. Item-level Content Validity Index (I-CVIs (item-level content validity index) were calculated for each item, S-CVI/Ave (average of the item-level content validity indices across items) was obtained as the mean of the item-level relevance CVIs, and modified kappa was computed, with decision thresholds pre-specified as I-CVI ≥ 0.83 (for N = 6) and S-CVI/Ave ≥ 0.90.

### 2.4. Statistical Analysis

Analyses used R 4.5.1 (R Foundation for Statistical Computing, Vienna, Austria). Because PEACE items are ordered categorical, dimensionality and reliability analyses were based primarily on polychoric correlations and ordinal latent-variable methods. Polychoric matrices were estimated using a two-step maximum-likelihood approach; if a matrix was not positive-definite, a near-positive-definite adjustment was applied before factor analysis [15,16].

We used a prespecified split-sample design to limit overfitting and allow independent exploratory and confirmatory steps. The overall target sample (N = 312) was planned a priori, with n = 125 (about 40%) allocated to EFA and n = 187 (about 60%) to CFA, consistent with recommendations for short, overdetermined factor models in EFA and small single-factor SEMs [17,18]. To obtain comparable subsamples, participants were stratified by sex, age (<40 vs. ≥40 years) and education (lower vs. high-school diploma/degree), and then randomly allocated within strata using a reproducible 40/60 split (set.seed(20251005)).

Construct validity and dimensionality were first examined in the EFA subsample. Factorability was assessed with the Kaiser–Meyer–Olkin (KMO) statistic—reported overall and at item level—and Bartlett’s test of sphericity [19,20]. Factors were extracted from the polychoric matrix using minimum residual (MINRES) with oblimin rotation. Factor retention was determined primarily by ordinal parallel analysis (MRFA-based) and Velicer’s minimum average partial (MAP) test; the Kaiser eigenvalue-greater-than-one rule was inspected descriptively but not used for decisions [21,22]. We report pattern loadings, communalities and RMSR.

In the CFA subsample, we fit a one-factor ordinal CFA model using WLSMV with probit thresholds, delta (δ) parameterization and std.lv = TRUE, treating PEACE-1 to PEACE-5 as ordered indicators [23,24]. We used δ as the primary single-group specification because, with five ordered indicators, the analogous theta (θ) model is exactly identified (df = 0), whereas the δ model remains testable (df = 5) [23,24]. No residual covariances were freed in the primary model.

Global CFA fit was summarized with unscaled and robust/scaled χ^2^, CFI, TLI, RMSEA with 90% confidence interval, and SRMR, with interpretation based primarily on the robust indices. Because RMSEA can be inflated in very low-df ordinal models, it was interpreted jointly with CFI/TLI/SRMR and local diagnostics [24]. Local fit was examined using standardized residual correlations and modification indices. One prespecified sensitivity model freed the residual covariance between sleep duration and subjective sleep quality. As a descriptive check of essential unidimensionality, we also fit a correlated two-factor model contrasting initiation/quantity items with maintenance items.

To assess robustness of the duration item without altering the primary specification, we repeated the one-factor CFA after (i) dichotomizing sleep duration as 7–8 h versus other durations, (ii) collapsing duration to three ordered bands (<6 h, 6–8 h, >8 h), and (iii) excluding the duration item. Single-group EFA and CFA were performed on complete cases; PEACE items had 0% item-level missingness, so all split-sample observations were retained.

Reliability was summarized with ordinal alpha calculated from the polychoric matrix, McDonald’s ω_total, and CFA-based composite reliability (Raykov’s ρc); 95% confidence intervals for alpha and omega were obtained with 1000 bootstrap resamples [25,26,27,28].

Measurement invariance by sex (women vs. men) was tested using multigroup ordered CFA under WLSMV with theta (θ) parameterization and Wu–Estabrook identification, following the sequence configural → thresholds → scalar (thresholds + loadings) [29]. For invariance models, the rarest upper response category was symmetrically collapsed across groups when needed to avoid sparse cells. Decisions were based primarily on WLSMV DIFFTEST, with changes in CFI, RMSEA and SRMR used as supportive evidence [30,31]. Item-level fairness was further examined with lordif using anchor purification at α = 0.01 and ΔMcFadden pseudo-R^2^ as an effect-size complement [32].

Validity testing followed a latent-first plan. Convergent validity was examined with a two-factor CFA relating PEACE to WHO-5 and, secondarily, with Spearman correlations between observed total scores. Latent convergent validity with WHO-5 was considered supportive when the lower bound of the 90% confidence interval for the latent correlation reached a pre-specified smallest effect size of interest (SESOI) of 0.15. Observed-score correlations were reported with 90% bootstrap confidence intervals [27] and interpreted against pre-specified direction/corridor rules (positive for WHO-5; for fatigue, related-but-distinct if the expected negative association was observed and |ρ| lay within 0.15–0.45). Discriminant/non-redundancy versus fatigue was assessed with Spearman correlations between PEACE and REST. Known-groups validity was evaluated with a MIMIC model regressing latent PEACE on BMI and sex [33]. Incremental validity for WHO-5 compared a baseline model including BMI and sex with a model additionally including PEACE; added value was considered supportive when the robust Satorra–Bentler χ^2^ difference was significant, and ΔR^2^ was at least 0.01 [30].

During manuscript preparation, ChatGPT (OpenAI, 5.2) was used only for language editing and for reviewing selected sections of the R code for clarity and error checking; it was not used to generate data, perform the statistical analyses, or interpret the results, and all outputs and final decisions were verified by the authors.

## 3. Results

### 3.1. Content Validity

Across Wave 1 (n = 10) cognitive interviews, participants paraphrased items as intended with no high-severity issues. Practical saturation (≥3 consecutive interviews without new meaning-level problems) was achieved within the first wave, and Wave 2 (n = 10) confirmed the absence of residual problems. No adverse events or need for assistance were noted; wording, recall period and response options were unchanged.

All six experts rated each item ≥ 3 (on 1–4 scales) for relevance, clarity and simplicity, yielding I-CVI and modified kappa = 1.00 for every item, with S-CVI/Ave = 1.00, exceeding pre-specified thresholds (I-CVI ≥ 0.83; S-CVI/Ave ≥ 0.90). These findings support initial content validity.

### 3.2. Sample Characteristics

Between December 2024 and June 2025, 312 community-dwelling adults were recruited via clinician-mediated invitations in private practices (general practitioners and specialists). After accrual closed, the cohort was partitioned as planned into CFA (n = 187; 59.9%) and EFA (n = 125; 40.1%) subsets.

Women represented 61.1% of the sample (61.5% CFA; 60.5% EFA). Age strata were balanced across decades (36.3%, 31.8%, 23.5% and 8.4% in increasing age categories), and 85.2% had at least a high-school diploma or degree. More than half reported no chronic disease (56.1%); the most prevalent conditions were dyslipidaemia (25.3%) and hypertension (21.2%), followed by renal stones (7.1%), autoimmune diseases (5.1%) and diabetes (3.2%). Cardiovascular events were uncommon (angina 1.0%, myocardial infarction 1.6%, stroke 0.3%); the ‘Prefer not to answer’ category for comorbidities accounted for 0.6%, with no missing values. Any prior cancer was reported by 10.6%, with small and similar site frequencies across splits. Apart from renal stones (CFA 1.6% vs. EFA 15.2%), the distributions were comparable between subsets.

PEACE items showed full category use. PEACE-1 (sleep duration) and PEACE-2 (subjective quality) concentrated in mid categories (1–2 ≈ 60–66%) with very few 4 s; PEACE-3 (sleep-onset difficulty) was more dispersed with substantial proportions in categories 1 (~25%) and 4 (~24%); PEACE-4 (night awakenings) was skewed toward the lower-score/worse end (category 0, corresponding to waking during the night three or more times a week, accounted for 33%); PEACE-5 (early-morning awakening) was more balanced and slightly right-skewed (4 ≈ 24%). Patterns were broadly similar across EFA and CFA subsets.

In this community sample, the PEACE total score had a median of 9 (IQR 6–13) on the 0–20 scale, indicating intermediate perceived sleep quality with ample variation. REST fatigue scores were similarly dispersed (median 12, IQR 7–22). WHO-5 scores centred around 14 (IQR 11–17) on the 0–25 metric (approx. 56, IQR 44–68 on the 0–100 scale), with the lower quartile falling below the standard < 13/25 cut-off for poor well-being. Distributions of PEACE, WHO-5, REST and BMI were essentially indistinguishable between CFA and EFA subsets. Extended baseline characteristics and item distributions are reported in Supplementary Tables S1–S6.

### 3.3. Construct Validity and Dimensionality

In the EFA subsample, the polychoric correlation matrix showed acceptable sampling adequacy (overall KMO = 0.76; item MSAs 0.72–0.87), and Bartlett’s test confirmed factorability (χ^2^(10) = 228.89, *p* < 0.001). Parallel analysis and MAP both indicated a single factor. A one-factor MINRES solution with oblimin rotation yielded homogeneous loadings (0.586–0.756) and communalities of 0.343–0.572, explaining 48.5% of common variance. Residual misfit was modest (RMSR = 0.095; df-corrected = 0.135). Detailed EFA diagnostics and factor-retention outputs are reported in Supplementary Tables S7–S12.

In the CFA subsample, a one-factor ordinal CFA (WLSMV) for the 0–4 PEACE items showed mixed but interpretable fit in this very low-df model: χ^2^_scaled(5) = 16.27, *p* = 0.006; CFI_scaled = 0.97; TLI_scaled = 0.94; RMSEA_scaled = 0.11 (90% CI 0.05–0.17); SRMR = 0.05. Standardized loadings were: subjective sleep quality 0.85 (R^2^ = 0.73), early-morning awakening 0.66 (R^2^ = 0.43), night-time awakenings 0.63 (R^2^ = 0.40), sleep duration 0.56 (R^2^ = 0.31) and sleep-onset difficulty 0.50 (R^2^ = 0.25). Local diagnostics indicated a modest residual covariance between duration and subjective quality, which is clinically plausible. A correlated two-factor solution (sleep adequacy/initiating sleep vs. sleep continuity) fit well and supported essential unidimensionality. Results were robust to alternative codings of the duration item. Detailed CFA fit indices, thresholds, local diagnostics, sensitivity analyses and duration-robustness results are reported in Supplementary Tables S13–S19.

Overall, EFA and CFA support essential unidimensionality interpretation, while also suggesting some localized dependence between duration and subjective quality. Standardized EFA and CFA loadings are summarized in Table 2.

### 3.4. Reliability and Measurement Invariance

Ordinal alpha for PEACE was 0.767 (95% CI 0.688–0.822), McDonald’s ω_total was 0.774 (95% CI 0.708–0.829), and composite reliability was 0.780. These indices indicate acceptable internal consistency for a brief five-item scale [25,26,27,28]. Reliability estimates are summarized in Table 3.

Measurement invariance by sex was broadly supported in multigroup ordinal CFA (women vs. men). Configural, threshold and scalar models all fit adequately, and DIFFTEST comparisons between thresholds vs. configural and scalar vs. thresholds were non-significant. However, at the scalar-versus-threshold step, ΔCFI was +0.015, indicating slightly higher CFI in the constrained model; DIFFTEST was non-significant, and ΔRMSEA and ΔSRMR remained favourable [30,31]. Item-level DIF analyses flagged no items after anchor purification [32]. Taken together, these findings support cautious comparison of PEACE scores between women and men without evidence of item-level bias. Measurement invariance results are summarized in Table 4, with full invariance and DIF outputs in Supplementary Tables S20–S22.

### 3.5. Associations with Well-Being and Fatigue; Known-Groups and Incremental Validity

Within a clinically oriented outcome framework, PEACE showed the expected pattern of associations with WHO-5 well-being and fatigue. At the latent level, a two-factor CFA revealed a positive correlation between the PEACE factor and the WHO-5 factor (φ = 0.303; 90% CI 0.184–0.422), comfortably exceeding a minimum-effect threshold while leaving substantial variance in WHO-5 to be explained by other determinants. On observed scores, PEACE total correlated positively with WHO-5 total (ρ = 0.271; 90% CI 0.151–0.383), indicating that better perceived sleep quality is associated with better mental well-being but is not redundant with it. Detailed latent and observed association results are reported in Supplementary Tables S23 and S24.

The association with REST fatigue was moderate and negative (ρ = −0.299; 90% CI −0.407 to −0.189), consistent with the notion that PEACE captures a sleep-specific construct linked to, but not interchangeable with, perceived tiredness.

Known-groups analysis aligned with clinical expectations: in a MIMIC model with latent PEACE as the outcome, higher BMI predicted lower sleep quality (standardized β = −0.301, *p* < 0.001), whereas sex showed no clear effect (standardized β = 0.017, *p* = 0.838).

Most importantly, from a general-medicine perspective, PEACE added non-trivial explanatory power for WHO-5 well-being beyond simple covariates. Adding PEACE to a model predicting WHO-5 from BMI and sex increased explained variance from R^2^ = 0.040 to R^2^ = 0.115 (ΔR^2^ = 0.075) and significantly improved model fit. Thus, a very short sleep-quality score accounted for an additional ~7.5% of the variance in mental well-being over and above BMI and sex, supporting the clinical relevance of perceived sleep quality and everyday well-being. Known-groups and incremental validity model outputs are reported in Supplementary Tables S25–S27.

## 4. Discussion

In this community-based sample of adults not selected for sleep disorders, the PEACE scale emerged as a brief and clinically feasible tool for assessing self-reported sleep quality. Content development, cognitive interviewing and expert ratings provided strong initial content validity evidence [34], and the psychometric analyses supported essential unidimensionality and satisfactory reliability. At the same time, the primary one-factor CFA showed mixed fit indices, and a modest residual association between duration and subjective quality suggests some localized dependence. The scale also performed similarly in men and women, with DIFFTEST-based support for measurement invariance and no evidence of DIF; at the scalar step, ΔCFI was +0.015, indicating slightly higher CFI in the constrained model, while ΔRMSEA and ΔSRMR remained favourable, so comparisons are best viewed as broadly supported rather than definitive.

From a clinical and general-medicine standpoint, a key question is how sleep quality, as measured by PEACE, relates to mental well-being and fatigue. In our sample, higher PEACE scores were consistently associated with better WHO-5 well-being and lower fatigue, at both latent and observed levels. The magnitude of these associations—small to moderate—fits the view that sleep is one important determinant of subjective well-being and energy, but not a proxy for generic outcomes. At the same time, the related-but-distinct pattern with fatigue supports the interpretation that PEACE is not merely a tiredness scale but taps into a broader construct of sleep continuity, adequacy and subjective restfulness.

The incremental validity results further underline the clinical interest of PEACE. Even in this routine-care community sample not receiving active treatment for major disease, adding a five-item sleep-quality score almost tripled the variance in WHO-5 well-being explained by a simple model based on BMI and sex. This suggests that, in general-practice and epidemiological contexts, a very short sleep PROM can capture meaningful variance in mental well-being that would otherwise be missed if only basic risk factors were considered.

When compared with established sleep questionnaires, PEACE appears to achieve a pragmatic balance between brevity and psychometric performance. The PSQI is widely used but relatively long and component-based [6]. The Sleep Condition Indicator and the Insomnia Severity Index are shorter and psychometrically well established, but they are more explicitly framed around insomnia [9,11]. PEACE, by contrast, uses five items to capture sleep duration, subjective quality and continuity in a format that can be co-administered easily with generic outcome measures.

The study has several strengths relevant to general medicine and primary care. First, the development and evaluation of PEACE followed contemporary PROM standards, with systematic content validation, ordinal factor-analytic methods, and explicit testing of invariance and DIF [12,13,23,24,29,30,31,32]. Second, the prespecified split-sample design reduced capitalization on chance and allowed independent exploratory and confirmatory testing [17,18]. Third, the inclusion of both WHO-5 and a fatigue scale allowed us to map PEACE onto clinically meaningful domains that are directly relevant to mood, functioning and everyday performance.

At the same time, several limitations should be acknowledged. The cross-sectional design does not permit causal inference regarding the direction of the associations between sleep, well-being and fatigue. Poor sleep may worsen mood and energy, but the reverse is also plausible, and shared factors such as chronic stress or pain could drive all three. Longitudinal and intervention studies are needed to clarify temporal dynamics and responsiveness. All study measures were self-reported; we did not include objective sleep metrics (e.g., actigraphy) or clinician-rated outcomes, so the link between PEACE scores and objective sleep architecture or clinically diagnosed sleep disorders remains to be established. In addition, because invitations were delivered opportunistically during routine consultations, we could not estimate the number approached or a formal response rate, which may have introduced selection uncertainty.

Furthermore, the sample consisted of Italian adults and excluded individuals under active treatment for major diseases. This was appropriate for an initial validation of a generic sleep PROM, but it limits generalisability to other cultural contexts, age groups, and clinical populations, such as patients with established insomnia, psychiatric disorders or chronic somatic illnesses.

Interpretability is another area where further work is needed. Unlike the PSQI and ISI, which have widely used cut-offs for poor sleep or clinically relevant insomnia [6,11], PEACE currently lacks validated thresholds for classifying severity levels or defining a minimal important change. In this first validation, we therefore focused on continuous scores and their associations with well-being and fatigue. Future research should combine anchor-based and distribution-based approaches to derive preliminary cut-off values and change estimates, for example, by linking PEACE to clinical diagnoses, patient-reported global ratings of change or established insomnia measures such as the PSQI and ISI [6,11].

Another priority is to examine the performance of PEACE in more diverse and clinically enriched settings. Cross-cultural validation and measurement-invariance testing in other languages and healthcare systems would determine whether the five-item structure and item hierarchy hold beyond this Italian sample. Studies in disease-specific cohorts (e.g., diabetes, cardiovascular disease, cancer, chronic pain or mental-health conditions) could evaluate how PEACE behaves when sleep is embedded within complex multimorbidity profiles and whether sleep quality mediates or moderates treatment effects on broader outcomes.

Finally, PEACE should be tested in the context of lifestyle-oriented and preventive interventions, where sleep is considered alongside physical activity, diet, weight management and stress [2,4]. In such settings, a very brief sleep-quality scale that can be integrated into routine assessments may help clinicians and public-health practitioners identify individuals with suboptimal sleep, tailor counselling and monitor changes over time.

## 5. Conclusions

This study provides initial evidence that the five-item PEACE scale is a brief patient-reported measure of perceived sleep quality that can be embedded in community and primary-care research. In a community sample of adults not selected for major disease, PEACE showed evidence consistent with essential unidimensionality, acceptable reliability, and supportive measurement invariance by sex. Its pattern of associations with fatigue and, crucially, with WHO-5 positive mental well-being suggests that PEACE captures a sleep-specific construct that is meaningfully related to, but not redundant with, broader patient-centred outcomes.

The finding that PEACE explained additional variance in WHO-5 well-being over and above BMI and sex highlights the contribution of perceived sleep quality to everyday functioning and mood, even in individuals not receiving active treatment. Given its brevity and straightforward scoring, PEACE may be useful for inclusion in community and primary-care surveys, clinical registries and intervention studies where questionnaire space is limited but sleep is a relevant determinant of outcomes. Further longitudinal, cross-cultural and ISI interpretative thresholds, responsiveness and links with objective sleep measures remain to be established.

## Figures and Tables

**Table 1:** Specification of Promoting Evaluation and Scoring Scheme.

Item ID	Canonical Short Label	Exact Question (EN Literal)	What It Captures	Response Coding (0 → 4)
PEACE-1	Sleep duration	On average, how many hours do you sleep each night?	Quantity of nightly sleep	0 = Less than 5 h; 1 = Between 5 and 6 h; 2 = Between 6 and 7 h; 3 = Between 7 and 8 h; 4 = More than 8 h.
PEACE-2	Subjective sleep quality	From a subjective point of view, how do you judge the quality of your sleep?	Global perceived sleep quality.	0 = very poor; 1 = mediocre; 2 = sufficient; 3 = good; 4 = excellent.
PEACE-3	Sleep onset difficulty	How often do you have difficulty falling asleep?	Problems initiating sleep	0 = Three or more times a week; 1 = Once or twice a week; 2 = Less than once a week; 3 = Less than once a month; 4 = Never.
PEACE-4	Night-time awakenings	How often do you wake up during the night?	Sleep maintenance.	0 = Three or more times a week; 1 = Once or twice a week; 2 = Less than once a week; 3 = Less than once a month; 4 = Never.
PEACE-5	Early-morning awakening	How often do you wake up too early and cannot fall back asleep?	Terminal insomnia	0 = Three or more times a week; 1 = Once or twice a week; 2 = Less than once a week; 3 = Less than once a month; 4 = Never.

**Table 2:** PEACE Items: EFA and CFA Standardized Loadings (λ = EFA/CFA; 2 dp).

ID	λ (EFA/CFA)
PEACE-1	0.59/0.56
PEACE-2	0.73/0.85
PEACE-3	0.70/0.50
PEACE-4	0.70/0.63
PEACE-5	0.76/0.66

Note: KMO = 0.76; Bartlett χ^2^(10) = 228.89, *p* < 0.001. CFA(δ,scaled): χ^2^s(5) = 16.27, *p* = 0.006; CFIs = 0.97; TLIs = 0.94; RMSEAs = 0.11 [0.05, 0.17]; SRMR = 0.05. In this very low-df setting, fit is interpreted cautiously and alongside the sensitivity model. Sens(PEACE1-PEACE2 freed): χ^2^s(4) = 8.45, *p* = 0.076; Δχ^2^s(1) = 7.09, *p* = 0.0078. Abbreviations: EFA, exploratory factor analysis; CFA, confirmatory factor analysis; λ, loading; KMO, Kaiser–Meyer–Olkin; CFI, comparative fit index; TLI, Tucker–Lewis index; RMSEA, root mean square error of approximation; SRMR, standardized root mean square residual; δ, delta parameterization; χ^2^s, scaled chi-square.

**Table 3:** Reliability (Polychoric-Based): Ordinal α (α_ord), ω, and Composite Reliability (CR).

α_ord	ω	CR
0.767 [0.688, 0.822]	0.774 [0.708, 0.829]	0.780

Abbreviations: α_ord, ordinal alpha; ω, omega; CR, composite reliability.

**Table 4:** Measurement Invariance by Sex (WLSMV, θ): DIFFTEST p and ΔCFI/ΔRMSEA/ΔSRMR.

Step	DIFF	ΔCFI	ΔRMSEA	ΔSRMR
Thresholds vs. Config	0.808	+0.004	−0.042	+0.000
Scalar vs. Thresholds	0.769	+0.015	−0.027	+0.000

Note: Δ = constrained − less-constrained; DIFF = WLSMV DIFFTEST p. DIFFTEST was non-significant at both steps; at the scalar step, ΔCFI = +0.015, indicating slightly higher CFI in the constrained model, while ΔRMSEA and ΔSRMR remained favourable. Abbreviations: WLSMV, weighted least squares means/variance adjusted; DIFFTEST, scaled χ^2^ diff-test; Δ, change; CFI, comparative fit index; RMSEA, root mean square error of approximation; SRMR, standardized root mean square residual; Config, configural; θ, theta parameterization.

## Data Availability

The individual-level dataset underlying this study contains health information that could risk re-identification and was collected under consent that did not permit open public release. The dataset is therefore not publicly available but can be provided upon reasonable request to the corresponding author.

## Supplementary Materials

The following supporting information can be downloaded at: https://www.mdpi.com/article/10.3390/medicina62040757/s1. Tables S1–S27, corresponding to the analyses described in the Methods and Results sections of the main manuscript, are provided in a separate Supplementary Materials file.
