# Supplementary material for: PEACE: Development and Validation of a Brief Five-Item Sleep Quality Scale for Community and Primary Care
Source: Medicina (Kaunas). 2026 Apr 15;62(4):757. doi: 10.3390/medicina62040757 (PMC13117621; doi:10.3390/medicina62040757)
Supplement: Supplementary file 1 [file medicina-62-00757-s001.zip › medicina-4228303-supplementary.pdf]

Supplementary Materials

PEACE: Development and Validation of a Brief Five-Item Sleep Quality Scale for Community and Primary Care

Table S1. Participant characteristics by analysis split (categorical variables): overall (N = 312), CFA (N = 187), and EFA (N = 125)

Variable	Category	Overall (N=312)	CFA (N=187)	EFA (N=125)
**Sex**	Female	**191 (61.1%)**	**115 (61.5%)**	**76 (60.5%)**
	Male	**121 (38.9%)**	**72 (38.5%)**	**49 (39.5%)**
**Age**	18–35	**114 (36.3%)**	**65 (34.8%)**	**49 (38.7%)**
	36–50	**99 (31.8%)**	**62 (33.2%)**	**37 (29.8%)**
	51–65	**73 (23.5%)**	**43 (23.0%)**	**30 (24.2%)**
	> 65	**26 (8.4%)**	**17 (9.1%)**	**9 (7.3%)**
**Education level**	Lower	**46 (14.8%)**	**28 (15.0%)**	**18 (14.5%)**
	High-school diploma or degree	**266 (85.2%)**	**159 (85.0%)**	**107 (85.5%)**

Table S2. Descriptive statistics for continuous measures (median [IQR]) by analysis split: overall (N = 312), CFA (N = 187), and EFA (N = 125)

Numeric Variable	Overall Median [IQR]	CFA Median [IQR]	EFA Median [IQR]
BMI	24.69 [22.12–27.59]	24.62 [22.17–27.78]	24.88 [22.06–27.22]
PEACE score	9 [6–13]	9 [6–12]	9 [5–13]
REST fatigue score	12 [7–22]	12 [7–22]	14 [7–23]

Numeric Variable	Overall Median [IQR]	CFA Median [IQR]	EFA Median [IQR]
WHO-5 well-being score	14 [11–17]	14 [10–17]	14 [11–17]

Table S3. Prevalence of self-reported diseases: overall (N = 312), CFA (N = 187), and EFA (N = 125)

Variable	Class	Overall % (n/312)	CFA % (n/187)	EFA % (n/125)
Diseases	**Missing**	0.0% (0)	0.0% (0)	0.0% (0)
Diseases	**Angina pectoris**	1.0% (3)	0.5% (1)	1.6% (2)
Diseases	**Gallstones (biliary stones)**	1.9% (6)	2.1% (4)	1.6% (2)
Diseases	**Kidney stones**	7.1% (22)	1.6% (3)	15.2% (19)
Diseases	**High cholesterol or triglycerides**	25.3% (79)	24.1% (45)	27.2% (34)
Diseases	**Diabetes**	3.2% (10)	3.2% (6)	3.2% (4)
Diseases	**Stroke**	0.3% (1)	0.5% (1)	0.0% (0)
Diseases	**Myocardial infarction (heart attack)**	1.6% (5)	1.6% (3)	1.6% (2)
Diseases	**Autoimmune disease**	5.1% (16)	6.4% (12)	3.2% (4)
Diseases	**Intestinal polyps**	2.2% (7)	2.7% (5)	1.6% (2)
Diseases	**Hypertension (high blood pressure)**	21.2% (66)	21.4% (40)	20.8% (26)
Diseases	**Gastric or duodenal ulcer**	1.6% (5)	1.6% (3)	1.6% (2)
Diseases	**None**	56.1% (175)	56.1% (105)	56.0% (70)
Diseases	**Prefer not to answer**	0.6% (2)	1.1% (2)	0.0% (0)

Table S4. Self-reported cancer history by type: overall (N = 312), CFA (N = 187), and EFA (N = 125)

Variable	Class	Overall % (n/312)	CFA % (n/187)	EFA % (n/125)
Cancer history types	**Missing**	0.0% (0)	0.0% (0)	0.0% (0)
Cancer history types	**Colorectal carcinoma**	2.2% (7)	2.7% (5)	1.6% (2)
Cancer history types	**Cervical carcinoma**	1.0% (3)	1.1% (2)	0.8% (1)
Cancer history types	**Prostate carcinoma**	1.9% (6)	1.6% (3)	2.4% (3)
Cancer history types	**Thyroid carcinoma**	0.3% (1)	0.5% (1)	0.0% (0)
Cancer history types	**Bladder carcinoma**	1.0% (3)	1.6% (3)	0.0% (0)
Cancer history types	**Hepatocellular carcinoma (liver)**	0.3% (1)	0.0% (0)	0.8% (1)
Cancer history types	**Breast carcinoma**	1.9% (6)	3.2% (6)	0.0% (0)
Cancer history types	**Ovarian carcinoma**	0.6% (2)	1.1% (2)	0.0% (0)
Cancer history types	**Renal cell carcinoma**	1.3% (4)	1.1% (2)	1.6% (2)
Cancer history types	**Leiomyosarcoma**	0.3% (1)	0.0% (0)	0.8% (1)
Cancer history types	**Non-Hodgkin lymphoma**	0.3% (1)	0.5% (1)	0.0% (0)
Cancer history types	**Melanoma**	0.6% (2)	0.0% (0)	1.6% (2)

Variable	Class	Overall % (n/312)	CFA % (n/187)	EFA % (n/125)
Cancer history types	**Multiple myeloma**	0.3% (1)	0.0% (0)	0.8% (1)
Cancer history types	**Brain tumors**	0.3% (1)	0.5% (1)	0.0% (0)
Cancer history types	**Malignant neoplasm (not listed)**	0.0% (0)	0.0% (0)	0.0% (0)

Table S5. Any self-reported cancer diagnosis (≥1 type): overall (N = 312), CFA (N = 187), and EFA (N = 125)

Variable	Class	Overall % (n/312)	CFA % (n/187)	EFA % (n/125)
Cancer history — ANY/meta	**Any cancer present (≥1 type)**	10.6% (33)	11.2% (21)	9.6% (12)
Cancer history — ANY/meta	**No cancer history**	89.1% (277)	88.8% (165)	89.6% (112)
Cancer history — ANY/meta	**Prefer not to answer**	0.6% (2)	0.5% (1)	0.8% (1)

Table S6. Response distribution for PEACE items 1–5 (0–4 scale): overall (N = 312), CFA (N = 187), and EFA (N = 125)

Variable	Class	OVERALL %	SPLIT=CFA %	SPLIT=EFA %
PEACE-1	0	11.3%	8.6%	15.3%
PEACE-1	1	29.3%	31.6%	25.8%
PEACE-1	2	37.3%	40.1%	33.1%
PEACE-1	3	21.9%	19.3%	25.8%

Variable	Class	OVERALL %	SPLIT=CFA %	SPLIT=EFA %
PEACE-1	4	0.3%	0.5%	0.0%
PEACE-2	0	7.1%	5.9%	8.9%
PEACE-2	1	30.9%	30.5%	31.5%
PEACE-2	2	35.4%	40.1%	28.2%
PEACE-2	3	23.5%	19.8%	29.0%
PEACE-2	4	3.2%	3.7%	2.4%
PEACE-3	0	18.6%	18.2%	19.4%
PEACE-3	1	25.1%	26.2%	23.4%
PEACE-3	2	17.4%	18.2%	16.1%
PEACE-3	3	15.1%	13.9%	16.9%
PEACE-3	4	23.8%	23.5%	24.2%
PEACE-4	0	33.1%	32.1%	34.7%
PEACE-4	1	28.0%	31.6%	22.6%
PEACE-4	2	14.8%	15.0%	14.5%

Variable	Class	OVERALL %	SPLIT=CFA %	SPLIT=EFA %
PEACE-4	3	13.2%	9.6%	18.5%
PEACE-4	4	10.9%	11.8%	9.7%
PEACE-5	0	21.2%	18.7%	25.0%
PEACE-5	1	19.9%	21.9%	16.9%
PEACE-5	2	13.2%	15.5%	9.7%
PEACE-5	3	21.5%	19.3%	25.0%
PEACE-5	4	24.1%	24.6%	23.4%

Table S7. Polychoric correlation settings and data diagnostics

Component	Setting / Finding
Correlation type	Polychoric (two-step ML)
Continuity correction	Applied (0.5)
Smoothing / near-PD check	cor.smooth fallback; Higham nearPD if needed
Warning: unequal categories	Yes — "items do not have an equal number of response alternatives"
Sparse cell adjustments	7 cells adjusted via continuity correction
Rotation (primary)	Oblimin
Extraction	MINRES (ULS)

Table S8. Sampling adequacy: overall KMO and item MSAs

Metric	Value
Overall KMO	0.76
MSA – PEACE1	0.72
MSA – PEACE2	0.75
MSA – PEACE3	0.87
MSA – PEACE4	0.74
MSA – PEACE5	0.76

Table S9. Bartlett's test of sphericity for the PEACE item correlation matrix

Statistic (χ^2)	df	p-value
228.8947	10	< 0.001

Table S10. Factor retention results from parallel analysis and MAP criteria

Method	Configuration	Suggested factors
Parallel Analysis (MRFA)	95th percentile; 1,000 datasets; polychoric	1
MAP (r^2)	Based on R (polychoric); N = complete cases	1
MAP (r^4)	Based on R (polychoric); N = complete cases	1

Table S11. Exploratory factor analysis: pattern loadings and communalities (h^2)

Item	Loading (F1)	h^2
PEACE-1	0.586	0.343
PEACE-2	0.727	0.529

Item	Loading (F1)	h²
PEACE-3	0.703	0.494
PEACE-4	0.697	0.485
PEACE-5	0.756	0.572

Table S12. Exploratory factor analysis summary: SS loadings, variance explained, and RMSR

Indicator	Value
SS loadings (F1)	2.424
Proportion variance explained	0.485
RMSR	0.0951
RMSR (df-corrected)	0.1345
Number of factors retained	1
Factor correlations (Φ)	Not applicable (single factor)

Table S13. Confirmatory factor analysis model fit indices (robust and unscaled) across specifications

Model	χ²	df	p	CFI	TLI	RMSEA [90% CI] (p-close)	SRMR	χ² (scaled)	df (scaled)	p (scaled)	CFI (scaled)	TLI (scaled)	RMSEA (scaled) [90% CI] (p-close)
Primary 1-factor	8.976	5	.110	.992	.985	.065 [.000–.133] (.295)	.050	16.273	5	.006	.970	.940	.110 [.053–.172] (.042)
Sensitivity (duration-quality residual covariance freed)	4.355	4	.360	.999	.998	.022 [.000–.115] (.577)	.034	8.450	4	.076	.988	.971	.077 [.000–.151] (.215)
3+2 correlated	2.512	4	.643	1.000	1.007	0 [.000–.089] (.803)	.027	4.835	4	.305	.998	.994	.033 [.000–.120] (.522)
Duration dichotomised	5.562	5	.351	.999	.997	.025 [.000–.107] (.594)	.053	9.383	5	.095	.986	.973	.069 [.000–.136] (.269)
Duration 3 bands	7.691	5	.174	.993	.987	.054 [.000–.124] (.393)	.053	12.626	5	.027	.976	.951	.091 [.028–.154] (.118)
No duration (4-item)	1.586	2	.453	1.000	1.004	0 [.000–.136] (.598)	.025	3.191	2	.203	.996	.987	.057 [.000–.167] (.345)

Table S14. Primary one-factor CFA: standardized factor loadings and item R^2

Item	λ (std.)	R^2
**Sleep duration**	.556	.309
**Sleep quality**	.852	.726
**Difficulty falling asleep**	.497	.247
**Night-time awakenings**	.634	.402
**Early morning awakening**	.659	.434

Table S15. Primary one-factor CFA: category thresholds (probit), standard errors, and Wald statistics

Outcome	Threshold	Probit (SE)	z	p
Sleep duration	t1	−1.369 (0.131)	−10.435	<.001
Sleep duration	t2	−0.251 (0.093)	−2.696	.007
Sleep duration	t3	0.849 (0.105)	8.087	<.001
Sleep duration	t4	2.553 (0.348)	7.327	<.001
Sleep quality	t1	−1.565 (0.147)	−10.637	<.001
Sleep quality	t2	−0.349 (0.094)	−3.712	<.001
Sleep quality	t3	0.722 (0.101)	7.134	<.001
Sleep quality	t4	1.781 (0.170)	10.449	<.001
Difficulty falling asleep	t1	−0.908 (0.107)	−8.482	<.001
Difficulty falling asleep	t2	−0.141 (0.092)	−1.531	.126
Difficulty falling asleep	t3	0.320 (0.094)	3.422	.001
Difficulty falling asleep	t4	0.722 (0.101)	7.134	<.001
Night-time awakenings	t1	−0.465 (0.096)	−4.867	<.001
Night-time awakenings	t2	0.349 (0.094)	3.712	<.001
Night-time awakenings	t3	0.793 (0.103)	7.683	<.001
Night-time awakenings	t4	1.187 (0.120)	9.910	<.001
Early morning awakening	t1	−0.888 (0.106)	−8.352	<.001
Early morning awakening	t2	−0.237 (0.093)	−2.550	.011
Early morning awakening	t3	0.155 (0.092)	1.677	.094
Early morning awakening	t4	0.687 (0.100)	6.856	<.001

Table S16. Primary one-factor CFA: local fit diagnostics (residual correlations, modification indices, EPC, and sepc.all)

Panel	Pair	Std. residual corr. (abs)	MI	EPC	sepc.all
A	Night-time awakenings with early morning awakening	+0.107			
A	Sleep duration with night-time awakenings	−0.098			
A	Sleep duration with early morning awakening	−0.096			

Panel	Pair	Std. residual corr. (abs)	MI	EPC	sepc.all
A	Sleep duration with sleep quality	+0.054			
A	Sleep quality with early morning awakening	−0.037			
A	Difficulty falling asleep with night-time awakenings	−0.029			
A	Sleep duration with difficulty falling asleep	+0.028			
A	Sleep quality with night-time awakenings	−0.019			
A	Sleep quality with difficulty falling asleep	−0.008			
A	Difficulty falling asleep with early morning awakening	+0.008			
B	Sleep duration with sleep quality		4.599	+0.200	+0.460
B	Sleep duration with early morning awakening		2.735	−0.146	−0.233
B	Sleep duration with night-time awakenings		2.618	−0.143	−0.222
B	Sleep duration with difficulty falling asleep		0.388	+0.044	+0.060

Table S17. Sensitivity model (sleep-duration/sleep-quality residual covariance freed)

Item	λ (std.)	R^2
**Sleep duration**	.447	.200
**Sleep quality**	.770	.593
**Difficulty falling asleep**	.522	.273
**Night-time awakenings**	.674	.454
**Early morning awakening**	.699	.488

Table S18. Correlated 3+2 model (InitQty vs Maint)

Factor	Item	λ (std.)	R^2
InitQty	**Sleep duration**	.556	.310
	**Sleep quality**	.918	.842

Factor	Item	λ (std.)	R^2
	**Difficulty falling asleep**	.497	.247
Maint	**Night-time awakenings**	.713	.509
	**Early morning awakening**	.736	.542

Table S19. Duration robustness — loadings across specifications

Model	Duration specification	λ(duration)	Other loadings (range)	Takeaway
Primary 1-factor	5 categories (0–4)	.556	.497–.852	Baseline; **robust** indices guide interpretation.
Dichotomised	7–8 h vs other	.564	.493–.813	Fit remains strong.
3 bands	<6 / 6–8 / >8 h	.468	.499–.820	Still solid; duration weakens slightly.
No duration	—	—	.492–.767	Core factor intact; fit remains strong.

Table S20. Measurement invariance by sex — robust fit indices (WLSMV, θ; ordered items)

Model step	CFI	RMSEA	SRMR
Configural	0.9584	0.1339	0.06243
Thresholds	0.9623	0.09235	0.06243
Scalar	0.9773	0.06512	0.06245

Table S21. Measurement invariance by sex — WLSMV DIFFTEST (decision rules shown)

Comparison	ΔCFI (signed)	ΔRMSEA (≤ .015)	ΔSRMR (≤ .010)	DIFFTEST p (ns to pass)	Decision
Thresholds vs Configural	+0.00399	−0.04152	+0.00000	0.8082	Supported (ns; CFI slightly higher in constrained model; deltas benign)
Scalar vs Thresholds	+0.01499	−0.02722	+0.00002	0.7685	Supportive but mixed overall (ns; CFI slightly higher in constrained model; |ΔCFI| slightly > .010)

Table S22. Item-level DIF by sex (lordif; α = .01; purification on)

Items tested	DIF items flagged	Criterion	α	Purification iters	Decision
PEACE1–PEACE5	0 / 5	Chi-square (McFadden pseudo-R^2)	0.01	1/10	PASS (no items flagged)

Table S23. Latent convergent validity — PEACE with WHO-5 (two-factor CFA)

Estimate	Value
Latent correlation φ	0.303
90% CI	[0.184, 0.422]
SESOI rule (LB90%(φ) ≥ 0.15)	PASS (LB90 = 0.184 ≥ 0.15)

Table S24. Observed convergent & discriminant validity (Spearman; 90% bootstrap CI)

Pair	ρ	90% CI (ρ)	90% CI |ρ|	Corridor [0.15, 0.45]	Direction rule	Decision
PEACE Total Score with WHO-5 Total Score	+0.271	[0.151, 0.383]	—	—	LB90(ρ) > 0 → Yes	Supports direction (secondary)
PEACE Total Score with REST Total Score (fatigue)	−0.299	[-0.407, -0.189]	[0.189, 0.407]	Inside → PASS	ρ < 0 → PASS	PASS (related-but-distinct)

Table S25. Known-groups validity (MIMIC): PEACE ~ BMI + sex (latent outcome; WLSMV, θ)

Predictor	Std. β (Std.all)	p-value	Expected direction	Decision
BMI	−0.301	< 0.001	Negative	Supportive
Sex (file coding)	+0.017	0.838	Pre-specified only	Inconclusive

Table S26. Incremental validity for WHO-5 (latent): adding PEACE beyond BMI + sex

Model	R^2(WHO-5)
M_0: WHO-5 ~ BMI + sex	0.0401
M_1: WHO-5 ~ BMI + sex + PEACE	0.1149
ΔR^2 (M_1−M_0)	+0.0748

Table S27. Incremental validity — model comparison (robust Satorra–Bentler χ^2 difference)

Comparison	robust SB $\Delta\chi^2$ p	Decision
M_1 vs M_0	0.000343	Supportive added value (significant; ΔR^2 non-trivial)

Abbreviations used in the supplementary tables: body mass index (BMI); confirmatory factor analysis (CFA); confidence interval (CI); comparative fit index (CFI); composite reliability (CR); differential item functioning (DIF); exploratory factor analysis (EFA); expected parameter change (EPC); interquartile range (IQR); Kaiser–Meyer–Olkin (KMO); measure(s) of sampling adequacy (MSA/MSAs); minimum average partial (MAP); minimum residual (MINRES); minimum rank factor analysis (MRFA); multiple-indicators multiple-causes (MIMIC); Recognizing and Estimating Signs of Tiredness (REST); root mean square error of approximation (RMSEA); Satorra–Bentler (SB); smallest effect size of interest (SESOI); standardized root mean square residual (SRMR); Tucker–Lewis index (TLI); weighted least squares mean- and variance-adjusted (WLSMV); WLSMV chi-square difference test (DIFFTEST); World Health Organization–Five Well-Being Index (WHO-5).
